# Supplementary material for: Early Colorectal Responses to HIV-1 and Modulation by Antiretroviral Drugs
Source: Vaccines (Basel). 2021 Mar 8;9(3):231. doi: 10.3390/vaccines9030231 (PMC8000905; doi:10.3390/vaccines9030231)
Supplement: Supplementary file 1 [file vaccines-09-00231-s001.zip › Supplementary Figure 4 new.pdf]

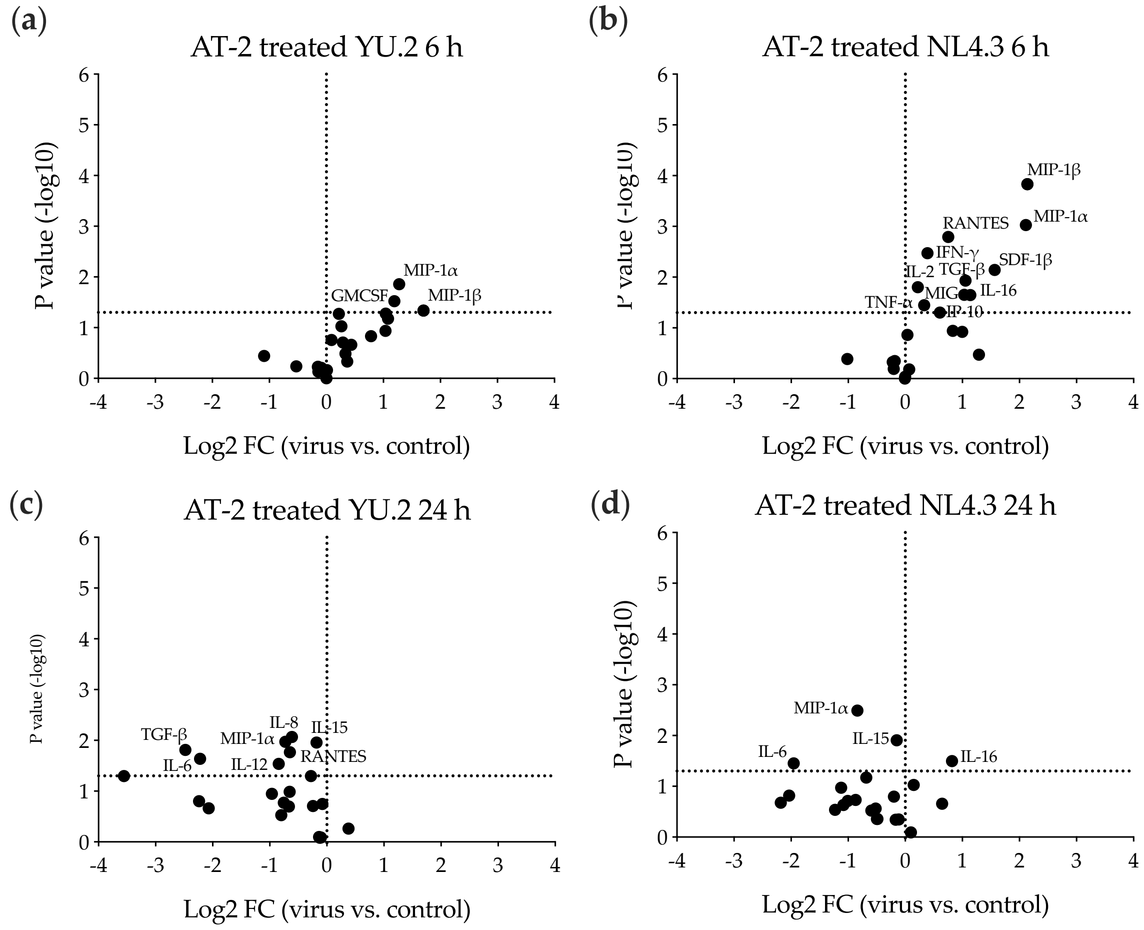

Figure S4. Modulation of colorectal cytokine profile induced by exposure to HIV-1. Volcano plots of all cytokines measured after 6 (**a**, **b**) or 24 h (**c**, **d**) of culture and comparing colorectal explants exposed to YU.2 (**a**, **c**) or NL4.3 (**b**, **d**) to those unchallenged (control). FC: fold change. Horizontal dotted line indicates a *P* value of 0.05. Analytes above this line are significantly modulated. Vertical dotted line is set at a $Log_2FC = 0$. Data shown are from quadruplicates with tissue from one donor.
