# Supplementary material for: Early Colorectal Responses to HIV-1 and Modulation by Antiretroviral Drugs
Source: Vaccines (Basel). 2021 Mar 8;9(3):231. doi: 10.3390/vaccines9030231 (PMC8000905; doi:10.3390/vaccines9030231)
Supplement: Supplementary file 1 [file vaccines-09-00231-s001.zip › Supplementary Figure 8.pdf]

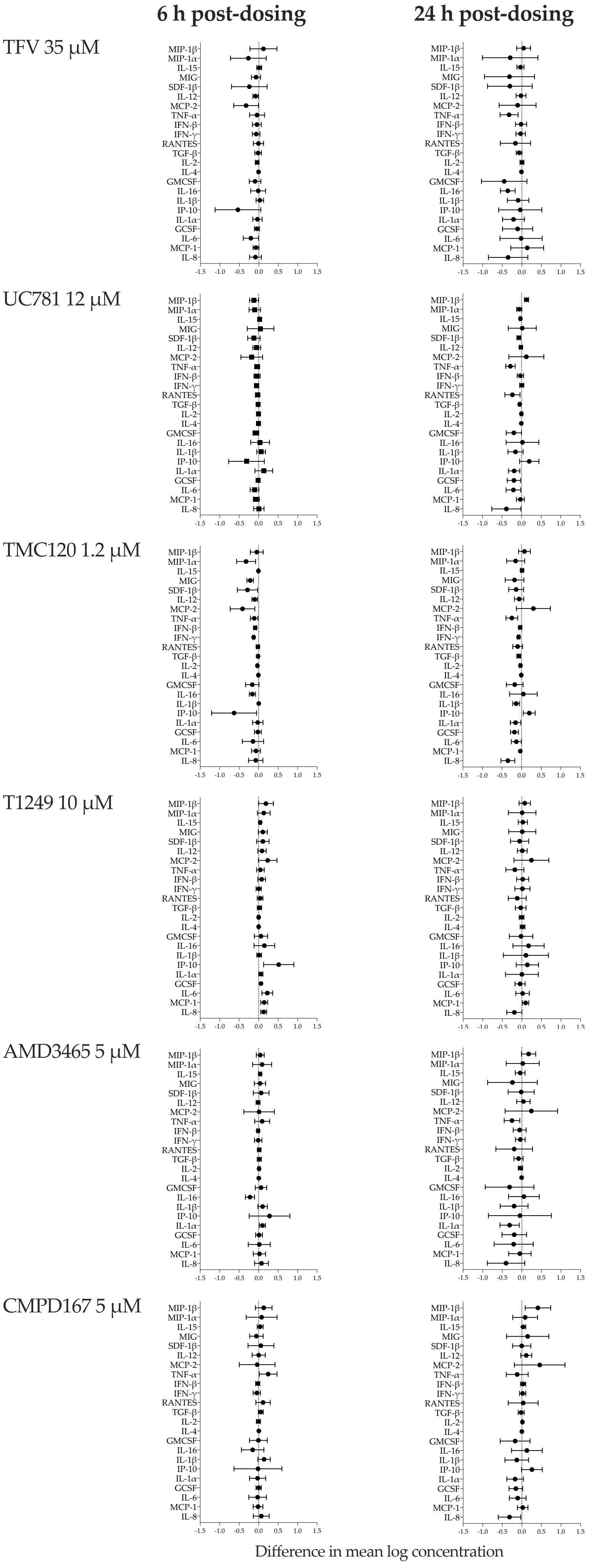

Figure S8. Effect of ARVs on the mucosal cytokine profile of colorectal explants. Difference of cytokine concentrations between *ex vivo* dosing with ARVs and non-treatment in mean log concentration ± SD from two independent experiments performed in quadruplicate.
