# Supplementary material for: Early Colorectal Responses to HIV-1 and Modulation by Antiretroviral Drugs
Source: Vaccines (Basel). 2021 Mar 8;9(3):231. doi: 10.3390/vaccines9030231 (PMC8000905; doi:10.3390/vaccines9030231)
Supplement: Supplementary file 1 [file vaccines-09-00231-s001.zip › Supplementary Figure 5.pdf]

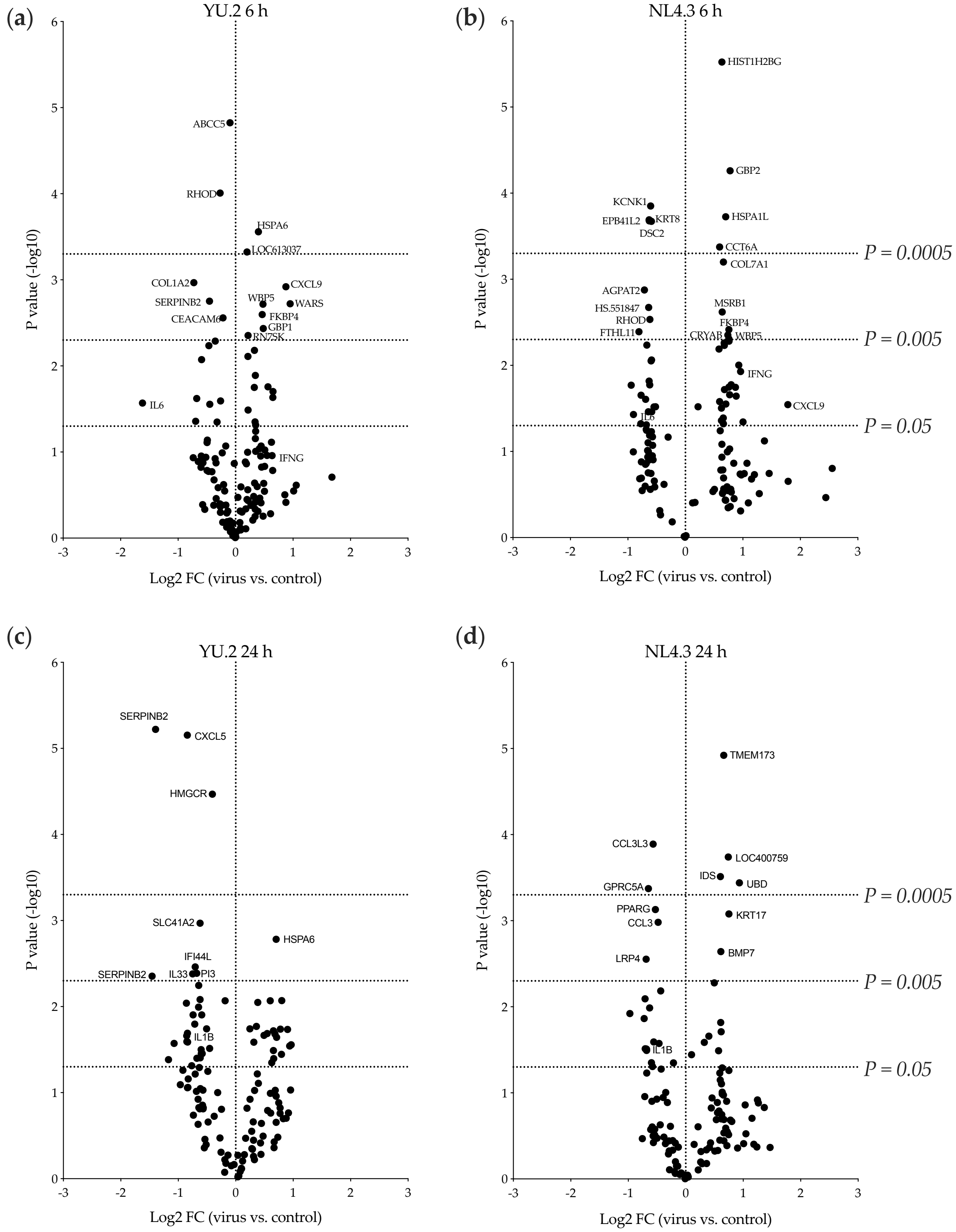

Figure S5. Modulation of colorectal transcriptomic profile induced by exposure to HIV-1. Volcano plots of all genes detected by RNA microarray after 6 (**a**, **b**) or 24 h (**c**, **d**) of culture and comparing colorectal explants exposed to YU.2 (**a**, **c**) or NL4.3 (**b**, **d**) to those unchallenged (control). FC: fold change. Vertical dotted line is set at a $Log_2FC = 0$. Data shown are from two independent experiments performed with tissue from different donors. Full list of statistically modulated genes can be found in Table S2.
