# Supplementary material for: Early Colorectal Responses to HIV-1 and Modulation by Antiretroviral Drugs
Source: Vaccines (Basel). 2021 Mar 8;9(3):231. doi: 10.3390/vaccines9030231 (PMC8000905; doi:10.3390/vaccines9030231)
Supplement: Supplementary file 1 [file vaccines-09-00231-s001.zip › Supplementary Figure 7.pdf]

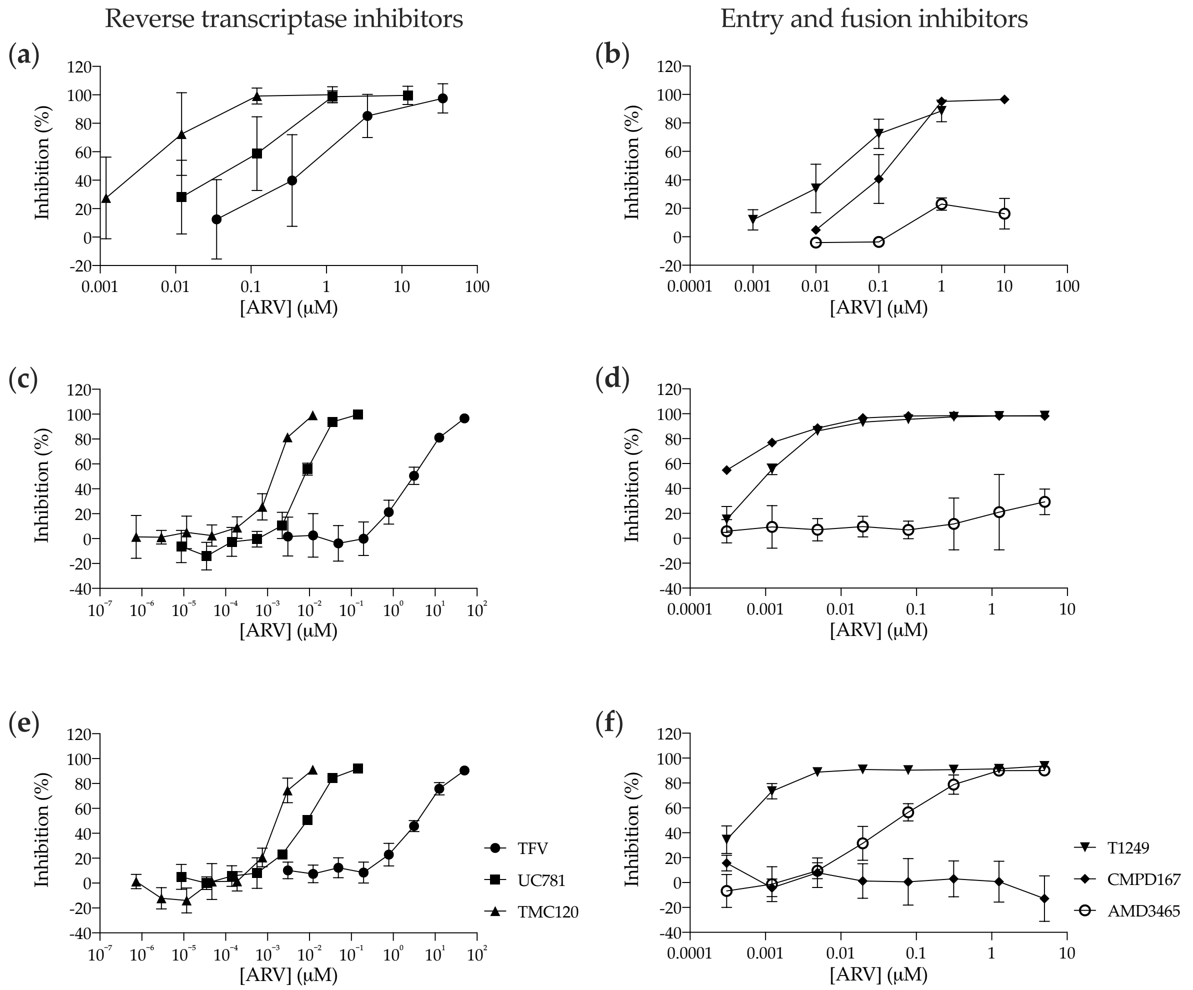

Figure S7. Activity of inhibitors against cYU.2 and cNL4.3 in colorectal explants and TZM-bl cells. Colorectal explants were treated for 1 h in the presence or absence of (**a**) TFV, UC781 or TMC120, or (**b**), T1249, CMPD167 or AMD3465 in serial dilutions. cYU.2 was added for 2 h before four washes with PBS. Explants were then transferred to gelfoam rafts and cultured for 15 days. The concentrations of p24 in the harvested supernatants were quantified by ELISA. TZM-bl cells were obtained from the NIH AIDS Research & Reference Reagent Program (http://www.aidsreagent.org/) and were grown in Dulbecco's Minimal Essential Medium (DMEM) (Sigma-Aldrich Inc., St Louis, Missouri, USA) containing 10% fetal calf serum (FCS), 2 mmol/l L-glutamine and antibiotics (100U of penicillin/ml, 100mg of streptomycin/ml). TZM-bl cells were treated for 1 h in the presence or absence of (**c**, **e**) TFV, UC781 or TMC120, or (**e**, **f**), T1249, CMPD167 or AMD3465 in serial dilutions. The cells were then exposed to cYU.2 (**c**, **d**) or cNL4.3 (**c**, **e**) (10^3 TCID$_{50}$). Luciferase expression (measured in relative light units) was determined after 48 h in cell lysates (Promega, Madison, Wisconsin, USA). The extent of inhibition by each drug was calculated. The percentage of inhibition was normalized to the p24 values obtained for explants or the relative light units obtained for cells not exposed to virus (0% infectivity) and for explants or cells infected with virus in the absence of drug (100% infectivity). Data are means (± SD) for triplicates of two colorectal specimens and for three independent TZM-bl assays performed in triplicate.
