# Supplementary material for: Early Colorectal Responses to HIV-1 and Modulation by Antiretroviral Drugs
Source: Vaccines (Basel). 2021 Mar 8;9(3):231. doi: 10.3390/vaccines9030231 (PMC8000905; doi:10.3390/vaccines9030231)
Supplement: Supplementary file 1 [file vaccines-09-00231-s001.zip › Supplementary Figure 6.pdf]

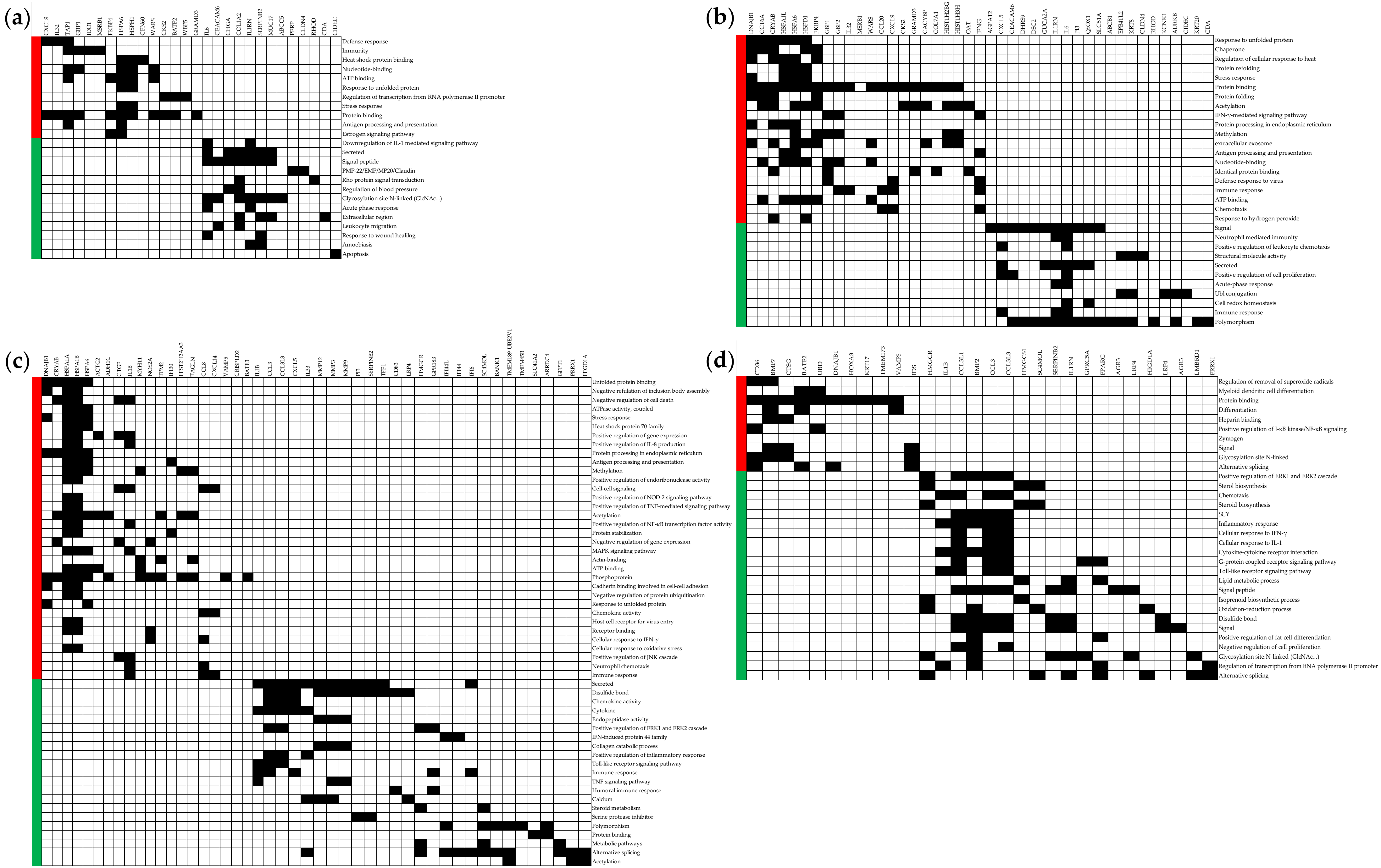

Figure S6. Biological processes associated with modulated genes in colorectal explants infected with HIV-1 YU.2 or NL4.3. RNA levels were measured in explants challenged with YU.2 (**a**, **c**) or NL4.3 (**b**, **d**) harvested 6 (**a**, **b**) or 24 h (**c**, **d**) post-challenge. Functional pathways were determined by DAVID 6.8 software for significantly up-regulated (red) or down-regulated (green) genes in infected explants compared to unchallenged control samples. Black squares represent analytes associated with pathway.
