# Supplementary material for: Early Colorectal Responses to HIV-1 and Modulation by Antiretroviral Drugs
Source: Vaccines (Basel). 2021 Mar 8;9(3):231. doi: 10.3390/vaccines9030231 (PMC8000905; doi:10.3390/vaccines9030231)
Supplement: Supplementary file 1 [file vaccines-09-00231-s001.zip › Supplementary Table 1.pdf]

Table S1. Concentrations of MIG and CCR5 chemokines secreted by colorectal explants infected with HIV-1 YU.2 or NL4.3.

Chemokine	Time post-challenge (h)	[Chemokine in culture supernatant][1] (pg/ml)	
		YU.2	NL4.3
MIG	6	10.78 ± 7.46	28.98 ± 14.58
	24	75.76 ± 32.97	189.80 ± 38.37
RANTES	6	0.01 ± 0.03	0.17 ± 0.02
	24	0.16 ± 0.09	0.45 ± 0.05
MIP-1α	6	0.61 ± 0.21	1.48 ± 0.44
	24	2.38 ± 0.41	3.63 ± 0.54
MIP-1β	6	1.13 ± 0.26	2.60 ± 0.66
	24	2.45 ± 0.85	4.12 ± 0.47

[1]Concentration value for each cytokine after subtraction of background value (average of concentrations in supernatants from unchallenged control tissue explants). Data are mean ± SD of four independent experiments performed with tissue from different donors.
