# Supplementary material for: Early Colorectal Responses to HIV-1 and Modulation by Antiretroviral Drugs
Source: Vaccines (Basel). 2021 Mar 8;9(3):231. doi: 10.3390/vaccines9030231 (PMC8000905; doi:10.3390/vaccines9030231)
Supplement: Supplementary file 1 [file vaccines-09-00231-s001.zip › Supplementary Table 2.pdf]

Table S2. List of significantly modulated genes by HIV-1 infection of colorectal explants[1].

YU.2, 6 h post-challenge

Gene	Definition	Virus vs. Ctrl FC	*P* value
GBP1	Homo sapiens guanylate binding protein 1, interferon-inducible, 67kDa	1.398	0.003676
IL6	Homo sapiens interleukin 6 (interferon, beta 2)	0.325	0.027
CIDEC	Homo sapiens cell death-inducing DFFA-like effector c	0.725	0.005806
BATF2	Homo sapiens basic leucine zipper transcription factor, ATF-like 2	1.569	0.023182
MSRB1	Homo sapiens selenoprotein X, 1 (SEPX1)	1.265	0.044604
FKBP4	Homo sapiens FK506 binding protein 4, 59kDa	1.380	0.002525
CXCL9	Homo sapiens chemokine (C-X-C motif) ligand 9	1.834	0.001204
IL32	Homo sapiens interleukin 32, transcript variant 4	1.271	0.01287
LOC613037	Homo sapiens nuclear pore complex interacting protein pseudogene on chromosome 16.	1.148	0.000475
CLDN4	Homo sapiens claudin 4 (CLDN4)	0.801	0.044791
SPSB2	Homo sapiens splA/ryanodine receptor domain and SOCS box containing 2	0.627	0.023888
HSPH1	Homo sapiens heat shock 105kDa/110kDa protein 1	1.162	0.03248
CKS2	Homo sapiens CDC28 protein kinase regulatory subunit 2	1.160	0.007754
MUC17	Homo sapiens mucin 17, cell surface associated	0.835	0.025487
SERPINB2	Homo sapiens serpin peptidase inhibitor, clade B (ovalbumin), member 2	0.730	0.001766
TAP1	Homo sapiens transporter 1, ATP-binding cassette, sub-family B (MDR/TAP)	1.571	0.019826
HSPA6	Homo sapiens heat shock 70kDa protein 6 (HSP70B')	1.318	0.000277
CHGA	Homo sapiens chromogranin A (parathyroid secretory protein 1)	0.664	0.008462
CEACAM6	Homo sapiens carcinoembryonic antigen-related cell adhesion molecule 6 (non-specific cross reacting antigen)	0.860	0.002765
ABCC5	Homo sapiens ATP-binding cassette, sub-family C (CFTR/MRP), member 5, transcript variant 1	0.935	0.000015
RHOD	Homo sapiens ras homolog gene family, member D	0.830	0.000098
PERP	Homo sapiens PERP, TP53 apoptosis effector	0.734	0.027909
IL1RN	Homo sapiens interleukin 1 receptor antagonist, transcript variant 4	0.618	0.04395
SNORD15B	Homo sapiens small nucleolar RNA, C/D box 15B on chromosome 11.	1.255	0.017743
CDA	Homo sapiens cytidine deaminase	0.784	0.005149
INDO	Homo sapiens indoleamine-pyrrole 2,3 dioxygenase	1.479	0.017511
HS.545589	Human small nuclear RNA U6atac, partial sequence	1.256	0.006587
GRAMD3	Homo sapiens GRAM domain containing 2B3	1.331	0.091592
COL1A2	Homo sapiens collagen, type I, alpha 2	0.605	0.001079
WARS	Homo sapiens tryptophanyl-tRNA synthetase, transcript variant 2	1.932	0.001899
WBP5/TCEAL9	Homo sapiens WW domain binding protein 5, transcript variant 2	1.392	0.001918
RN7SK	Homo sapiens RNA, 7SK small nuclear on chromosome 6.	1.164	0.004421

NL4.3, 6 h post-challenge

Gene	Definition	Virus vs. Ctrl FC	*P* value
KRT80	PREDICTED: similar to Keratin, type II cytoskeletal 8 (Cytokeratin-8) (CK-8)	0.664	0.000212
GBP1	Homo sapiens guanylate binding protein 1, interferon-inducible, 67kDa	1.846	0.022778
IL6	Homo sapiens interleukin 6 (interferon, beta 2)	0.535	0.037185
FTHL11	Homo sapiens ferritin, heavy polypeptide-like 11 on chromosome 8.	0.571	0.00406
LOC23117	PREDICTED: Homo sapiens KIAA0220-like protein, transcript variant 16	1.582	0.047665
HIST1H3H	Homo sapiens histone cluster 1, H3h	1.510	0.026331
CIDEC	Homo sapiens cell death-inducing DFFA-like effector c	0.584	0.047736
MSRB1	Homo sapiens selenoprotein X, 1 (SEPX1)	1.559	0.002401
AGPAT2	Homo sapiens 1-acylglycerol-3-phosphate O-acyltransferase 2 (lysophosphatidic acid acyltransferase, beta), transcript variant 2	0.611	0.001333
FKBP4	Homo sapiens FK506 binding protein 4, 59kDa	1.690	0.003843
CXCL9	Homo sapiens chemokine (C-X-C motif) ligand 9	3.438	0.028569
IL32	Homo sapiens interleukin 32, transcript variant 4,	1.604	0.019126
HSPA1L	Homo sapiens heat shock 70kDa protein 1-like	1.626	0.000188
KCNK1	Homo sapiens potassium channel, subfamily K, member 1	0.658	0.000141
CLDN4	Homo sapiens claudin 4	0.640	0.03462
KRT20	Homo sapiens keratin 20	0.645	0.015206
PI3	Homo sapiens peptidase inhibitor 3, skin-derived (SKALP)	0.665	0.008643
GUCA2A	Homo sapiens guanylate cyclase activator 2A (guanylin)	0.619	0.024824
C4ORF7	Homo sapiens chromosome 4 open reading frame 7	1.165	0.03018
QSOX1	Homo sapiens quiescin Q6 sulfhydryl oxidase 1, transcript variant 2	0.521	0.016957
HSPD1	Homo sapiens heat shock 60kDa protein 1 (chaperonin), nuclear gene encoding mitochondrial protein, transcript variant 1	1.580	0.040898
CCL20	Homo sapiens chemokine (C-C motif) ligand 20	1.737	0.016764
CXCL5	Homo sapiens chemokine (C-X-C motif) ligand 5	0.661	0.008863
CKS2	Homo sapiens CDC28 protein kinase regulatory subunit 2	1.596	0.005492
AURKB	Homo sapiens aurora kinase B	0.698	0.030258
LOC85389	Homo sapiens RNA, small nucleolar on chromosome 11.	1.601	0.005874
CACYBP	Homo sapiens calcyclin binding protein, transcript variant 1	1.690	0.017904
HSPA6	Homo sapiens heat shock 70kDa protein 6 (HSP70B’)	1.707	0.02195
CEACAM6	Homo sapiens carcinoembryonic antigen-related cell adhesion molecule 6	0.629	0.00582
HS.551847	601452348F1 NIH_MGC_66 Homo sapiens cDNA clone IMAGE:3856355 5	0.642	0.002128
DHRS9	Homo sapiens dehydrogenase/reductase (SDR family) member 9, transcript variant 1	0.667	0.034626
RHOD	Homo sapiens ras homolog gene family, member D	0.652	0.002925
IL1RN	Homo sapiens interleukin 1 receptor antagonist, transcript variant 4	0.685	0.030337
EPB41L2	Homo sapiens erythrocyte membrane protein band 4.1-like 2	0.646	0.000204
CDA	Homo sapiens cytidine deaminase	0.586	0.022231
TMEM17	Homo sapiens transmembrane protein 17	1.694	0.005234
COL7A1	Homo sapiens collagen, type VII, alpha 1 (epidermolysis bullosa, dystrophic, dominant and recessive)	1.582	0.00063
CCT6A	Homo sapiens chaperonin containing TCP1, subunit 6A, transcript variant 1	1.513	0.000422
LOC643300	PREDICTED: Homo sapiens similar to 60 kDa heat shock protein, mitochondrial precursor (Hsp60) (60 kDa chaperonin) (CPN60)	1.829	0.017949
GRAMD3	Homo sapiens GRAM domain containing 3	1.544	0.031316
DSC2	Homo sapiens desmocollin 2, transcript variant Dsc2b,	0.648	0.00021
HIST1H2BG	Homo sapiens histone cluster 1, H2bg	1.555	0.000003
CRYAB	Homo sapiens crystallin, alpha B	1.669	0.004446
WBP5/TCEAL9	Homo sapiens WW domain binding protein 5, transcript variant 2	1.691	0.004923
WARS	Homo sapiens tryptophanyl-tRNA synthetase, transcript variant 1	2.003	0.045382
GBP2	Homo sapiens guanylate binding protein 2, interferon-inducible	1.715	0.000055
DNAJB1	Homo sapiens DnaJ (Hsp40) homolog, subfamily B, member 1	1.905	0.009926
OAT	Homo sapiens ornithine aminotransferase (gyrate atrophy), nuclear gene encoding mitochondrial protein	1.546	0.043794
RN7SK	Homo sapiens RNA, 7SK small nuclear on chromosome 6.	1.501	0.006451
ABCB1	Homo sapiens ATP-binding cassette, sub-family B (MDR/TAP), member 1	0.650	0.016885
IFNG	Homo sapiens interferon, gamma	1.947	0.011766
OSTALPHA	Homo sapiens organic solute transporter alpha	0.625	0.04906

YU.2, 24 h post-challenge

Gene	Definition	Virus vs. Ctrl FC	*P* value
TPM2	Homo sapiens tropomyosin 2 (beta), transcript variant 2	1.545	0.044883
VAMP5	Homo sapiens vesicle-associated membrane protein 5 (myobrevin)	1.246	0.02593
HMGCR	Homo sapiens 3-hydroxy-3-methylglutaryl-Coenzyme A reductase	0.755	0.000034
BANK1	Homo sapiens B-cell scaffold protein with ankyrin repeats 1, transcript variant 2,	0.598	0.012496
TAGLN	Homo sapiens transgelin, transcript variant 2	1.577	0.032365
IFI6	Homo sapiens interferon, alpha-inducible protein 6, transcript variant 3	0.610	0.016017
CCL8	Homo sapiens chemokine (C-C motif) ligand 8	1.406	0.021607
CXCL14	Homo sapiens chemokine (C-X-C motif) ligand 14	1.717	0.018249
PRRX1	Homo sapiens paired related homeobox 1, transcript variant pmx-1a	0.639	0.010154
CTGF	Homo sapiens connective tissue growth factor	1.638	0.022762
PI3	Homo sapiens peptidase inhibitor 3, skin-derived (SKALP)	0.626	0.004103
MMP12	Homo sapiens matrix metallopeptidase 12 (macrophage elastase)	0.560	0.025793
IL1B	Homo sapiens interleukin 1, beta	0.554	0.02187
TFF1	Homo sapiens trefoil factor 1	0.661	0.035134
CXCL5	Homo sapiens chemokine (C-X-C motif) ligand 5	0.558	0.000007
GPR183	Homo sapiens Epstein-Barr virus induced gene 2 (lymphocyte-specific G protein-coupled receptor)	0.659	0.031629
MMP3	Homo sapiens matrix metallopeptidase 3 (stromelysin 1, progelatinase)	0.589	0.048596
CRISPLD2	Homo sapiens cysteine-rich secretory protein LCCL domain containing 2	1.581	0.040187
BATF3	Homo sapiens basic leucine zipper transcription factor, ATF-like 3	1.187	0.018133
GFPT1	Homo sapiens glutamine-fructose-6-phosphate transaminase 1	0.626	0.039843
ACTG2	Homo sapiens actin, gamma 2, smooth muscle, enteric	1.868	0.01845
CD83	Homo sapiens CD83 molecule, transcript variant 2	0.651	0.039298
NOS2A	Homo sapiens nitric oxide synthase 2A (inducible, hepatocytes), transcript variant 2	1.284	0.016988
ADH1C	Homo sapiens alcohol dehydrogenase 1C (class I), gamma polypeptide	1.604	0.020834
ARRDC4	Homo sapiens arrestin domain containing 4	0.667	0.035182
IFI30	Homo sapiens interferon, gamma-inducible protein 30	1.305	0.008927
HIGD1A	Homo sapiens HIG1 domain family, member 1A, transcript variant 1,	0.881	0.008539
TMEM189-UBE2V1	Homo sapiens TMEM189-UBE2V1, transcript variant 2,	0.640	0.00567
SERPINB2	Homo sapiens serpin peptidase inhibitor, clade B (ovalbumin), member 2	0.380	0.000006
HSPA6	Homo sapiens heat shock 70kDa protein 6 (HSP70B')	1.629	0.001648
TMEM45B	Homo sapiens transmembrane protein 45B	0.664	0.012473
LRP4	Homo sapiens low density lipoprotein receptor-related protein 4	0.704	0.018154
IFI6	Homo sapiens interferon, alpha-inducible protein 6, transcript variant 2,	0.476	0.026821
IL33	Homo sapiens interleukin 33	0.595	0.004163
SLC41A2	Homo sapiens solute carrier family 41, member 2	0.651	0.001072
HIST2H2AA3	Homo sapiens histone cluster 2, H2aa3	1.516	0.008546
IFI44L	Homo sapiens interferon-induced protein 44-like	0.613	0.003454
MYH11	Homo sapiens myosin, heavy chain 11, smooth muscle, transcript variant SM1A	1.737	0.035925
IFI44	Homo sapiens interferon-induced protein 44	0.651	0.008281
SC4MOL	Homo sapiens sterol-C4-methyl oxidase-like, transcript variant 1	0.730	0.030646
MMP9	Homo sapiens matrix metallopeptidase 9 (gelatinase B, 92kDa gelatinase, 92kDa type IV collagenase)	0.444	0.041349
CRYAB	Homo sapiens crystallin, alpha B	1.577	0.019085
CCL3	Homo sapiens chemokine (C-C motif) ligand 3	0.559	0.020373
HSPA1A	Homo sapiens heat shock 70kDa protein 1A	1.462	0.020599
CCL3L3	Homo sapiens chemokine (C-C motif) ligand 3-like 3	0.555	0.025479
DNAJB1	Homo sapiens DnaJ (Hsp40) homolog, subfamily B, member 1	1.744	0.008507
HSPA1B	Homo sapiens heat shock 70kDa protein 1B	1.919	0.028644

NL4.3, 24 h post-challenge

Gene	Definition	Virus vs. Ctrl FC	P value
VAMP5	Homo sapiens vesicle-associated membrane protein 5 (myobrevin)	1.533	0.019543
HMGCR	Homo sapiens 3-hydroxy-3-methylglutaryl-Coenzyme A reductase (	0.670	0.049481
BATF2	Homo sapiens basic leucine zipper transcription factor, ATF-like 2	1.527	0.015232
HMGCS1	Homo sapiens 3-hydroxy-3-methylglutaryl-Coenzyme A synthase 1 (soluble) transcript variant 2	0.647	0.010298
UBD	Homo sapiens ubiquitin D	1.910	0.000363
AGR3	Homo sapiens anterior gradient homolog 3 (Xenopus laevis)	0.624	0.031084
PRRX1	Homo sapiens paired related homeobox 1, transcript variant pmx-1a	0.740	0.006546
LOC400759	Homo sapiens similar to Interferon-induced guanylate-binding protein 1 (GTP-binding protein 1) (Guanine nucleotide-binding protein 1) (HuGBP-1) on chromosome 1.	1.672	0.000182
IL1B	Homo sapiens interleukin 1, beta	0.614	0.030705
LOC652493	PREDICTED: Homo sapiens similar to Ig kappa chain V-I region HK102 precursor	1.251	0.025879
GPRC5A	Homo sapiens G protein-coupled receptor, family C, group 5, member A	0.637	0.000424
PPARG	Homo sapiens peroxisome proliferator-activated receptor gamma, transcript variant 1	0.694	0.000742
HIGD1A	Homo sapiens HIG1 domain family, member 1A, transcript variant 1	0.624	0.032159
KRT17	Homo sapiens keratin 17	1.684	0.000834
SERPINB2	Homo sapiens serpin peptidase inhibitor, clade B (ovalbumin), member 2	0.510	0.01197
LRP4	Homo sapiens low density lipoprotein receptor-related protein 4	0.620	0.002798
CCL3L1	Homo sapiens chemokine (C-C motif) ligand 3-like 1	0.681	0.025654
CTSG	Homo sapiens cathepsin G	1.413	0.005265
IL1RN	Homo sapiens interleukin 1 receptor antagonist, transcript variant 4	0.863	0.044819
CD36	Homo sapiens CD36 molecule (thrombospondin receptor), transcript variant 3	1.072	0.036037
TMEM173	Homo sapiens transmembrane protein 173	1.583	0.000012
ATAD4	Homo sapiens ATPase family, AAA domain containing 4	0.612	0.008077
HOXA3	Homo sapiens homeobox A3, transcript variant 2	1.322	0.021947
IDS	Homo sapiens iduronate 2-sulfatase (Hunter syndrome), transcript variant 2,	1.519	0.000307
LMBRD1	Homo sapiens LMBR1 domain containing 1	0.621	0.030836
BMP2	Homo sapiens bone morphogenetic protein 2	0.662	0.044677
SC4MOL	Homo sapiens sterol-C4-methyl oxidase-like, transcript variant 1	0.606	0.013694
CCL3	Homo sapiens chemokine (C-C motif) ligand 3	0.717	0.001042
BMP7	Homo sapiens bone morphogenetic protein 7 (osteogenic protein 1)	1.529	0.002284
CCL3L3	Homo sapiens chemokine (C-C motif) ligand 3-like 3	0.674	0.000129
DNAJB1	Homo sapiens DnaJ (Hsp40) homolog, subfamily B, member 1	1.487	0.032428
VAMP5	Homo sapiens vesicle-associated membrane protein 5 (myobrevin)	1.533	0.019543

[1]Significantly modulated genes as determined by unpaired t test with a Benjemini-Hochberg multiple testing correction ($P < 0.05$)
FC: Fold change
