# Supplementary material for: Early Colorectal Responses to HIV-1 and Modulation by Antiretroviral Drugs
Source: Vaccines (Basel). 2021 Mar 8;9(3):231. doi: 10.3390/vaccines9030231 (PMC8000905; doi:10.3390/vaccines9030231)
Supplement: Supplementary file 1 [file vaccines-09-00231-s001.zip › Supplementary Figure 1.pdf]

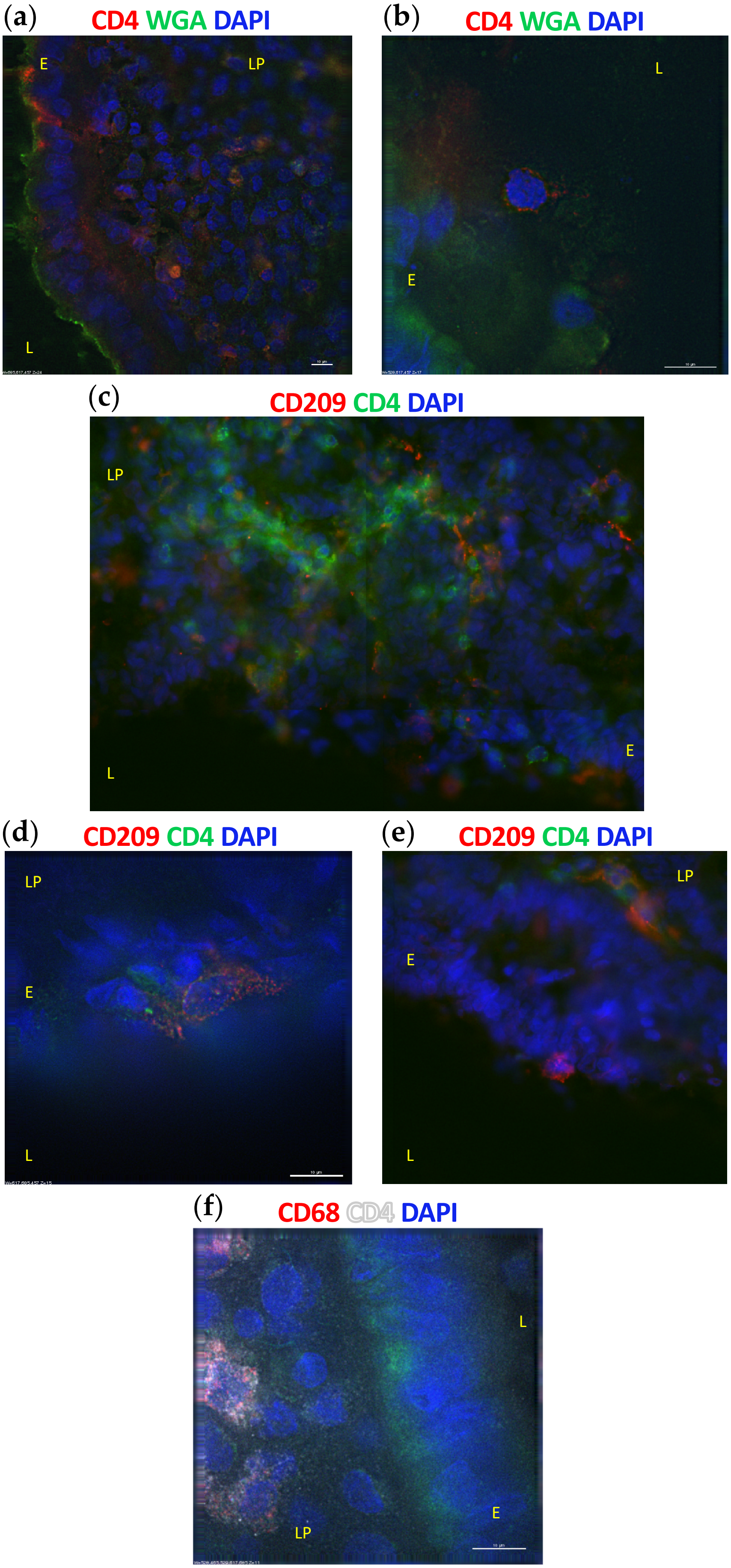

Figure S1. Tissue resident immune cells in colorectal tissue. Representative tissue cryosections immunofluorescently stained with (**a**, **b**) OKT4-RedX to detect CD4^{+} cells and WGA-Cy5 to label the columnar epithelium; (**c**, **d**, **e**) α-CD209-RedX and α-CD4-AlexaFluor647 antibodies to detect DCs and CD4^{+} cells, respectively; or (**f**) α-CD68-RedX and α-CD4-AlexaFluor647 antibodies to detect macrophages and CD4^{+} cells, respectively. Cell nuclei stained with DAPI (blue). White bar = 10 µm. L: lumen; E: epithelium; LP: lamina propia.
