# Supplementary material for: Early Colorectal Responses to HIV-1 and Modulation by Antiretroviral Drugs
Source: Vaccines (Basel). 2021 Mar 8;9(3):231. doi: 10.3390/vaccines9030231 (PMC8000905; doi:10.3390/vaccines9030231)
Supplement: Supplementary file 1 [file vaccines-09-00231-s001.zip › Supplementary Table 3.pdf]

Table S3. List of significantly modulated cytokines by dosing with ARVs and HIV-1 infection of colorectal explants[1].

NL4.3 ± ARV, 6 h post-treatment

	Treatment[1]													
	Virus		TFV 35 μM		UC781 12 μM		TMC120 1.2 μM		T1249 10 μM		AMD3465 5 μM		CMPD167 5 μM	
Cytokine	FC	*P*	FC	*P*	FC	*P*	FC	*P*	FC	*P*	FC	*P*	FC	*P*
IL-8	4.165	0.0564070	1.612	0.0179589	2.915	0.0060963	2.431	0.0001251	2.345	0.0671596	2.324	0.0010492	2.068	0.0007800
MCP-1	2.925	0.0612790	2.074	0.0157406	2.921	0.0127667	2.659	0.0294708	2.214	0.0558412	2.054	0.0350007	2.068	0.0316410
IL-6	1.103	0.8664470	0.855	0.7959987	1.088	0.8766056	0.983	0.9765724	0.897	0.8558390	0.834	0.7692537	0.893	0.8496004
GCSF	1.308	0.2485120	2.195	0.0004054	2.339	0.0002530	2.318	0.0003237	2.288	0.0004008	2.258	0.0003732	2.260	0.0003187
IL-1α	7.603	0.0002500	0.050	0.0014505	0.057	0.0015074	0.056	0.0015054	0.053	0.0014795	0.057	0.0015067	0.052	0.0014672
IP-10	12.937	0.0064490	35.688	0.0142703	67.598	0.0121893	54.921	0.0112898	34.320	0.0066698	27.174	0.0703082	24.470	0.0041638
IL-1β	1.964	0.0154460	0.763	0.1727533	0.905	0.5686441	1.014	0.9390240	0.917	0.6307745	1.145	0.4827313	0.861	0.4442053
IL-16	1.516	0.1731740	0.239	0.0197161	1.065	0.8862978	1.272	0.5864865	0.664	0.2402865	0.825	0.6211252	0.575	0.2146643
GMCSF	3.243	0.0050290	2.118	0.0641755	3.092	0.0168952	2.521	0.0117180	2.570	0.0121415	2.555	0.0144093	1.879	0.0940835
IL-4	1.092	0.0791320	1.025	0.7217409	1.043	0.7619075	1.072	0.2893904	0.960	0.7052923	1.011	0.9488090	0.889	0.3324874
IL-2	1.249	0.0025830	0.357	0.0000035	0.473	0.0005497	0.425	0.0000059	0.352	0.0000020	0.500	0.0006667	0.326	0.0000040
TGF-β	3.146	0.0031510	33.127	0.0000066	40.018	0.0000249	36.653	0.0000000	33.194	0.0000031	39.707	0.0000303	31.994	0.0000004
RANTES	2.046	0.0000270	2.798	0.0002539	4.757	0.0008884	3.248	0.0000742	2.827	0.0000014	3.480	0.0000238	3.486	0.0000480
IFN-γ	1.677	0.0019620	4.314	0.0000064	5.493	0.0000533	4.974	0.0000001	4.534	0.0000018	5.567	0.0000174	3.997	0.0000028
IFN-β	1.687	0.0196360	2.200	0.0853799	2.138	0.0056859	2.032	0.0035558	1.609	0.0090557	1.338	0.2883588	1.579	0.0492593
TNF-α	1.569	0.0001740	2.114	0.0000006	2.448	0.0000282	2.195	0.0000000	2.166	0.0000001	2.146	0.0000000	2.087	0.0000002
MCP-2	6.392	0.0133480	14.030	0.0034929	26.835	0.0149017	22.058	0.0280604	18.792	0.0017614	14.604	0.0220437	16.325	0.0126738
IL-12	1.179	0.0920770	3.764	0.0035546	4.182	0.0006017	3.874	0.0000182	3.478	0.0000933	3.610	0.0000029	3.126	0.0008171
SDF-1β	4.100	0.0003030	7.099	0.0000009	11.212	0.0100450	7.664	0.0000002	6.580	0.0057105	5.760	0.0081427	6.329	0.0099694
MIG	10.519	0.0076240	12.500	0.0341172	22.505	0.0066723	18.067	0.0056154	10.190	0.0034069	10.613	0.0304912	10.594	0.0001738
IL-15	1.069	0.0093050	0.858	0.0017124	0.899	0.0019073	0.877	0.0001101	0.876	0.0006005	0.902	0.0011307	0.865	0.0000755
MIP-1α	8.298	0.0006040	12.470	0.0000478	22.917	0.0091999	17.710	0.0022051	15.643	0.0008783	12.601	0.0070619	13.580	0.0017738
MIP-1β	7.603	0.0002500	4.731	0.0002807	5.995	0.0001527	6.460	0.0107440	6.578	0.0012100	5.585	0.0360662	4.437	0.0015248

YU.2 ± ARV, 6 h post-treatment														
	Treatment[1]													
	Virus		TFV 35 μM		UC781 12 μM		TMC120 1.2 μM		T1249 10 μM		AMD3465 5 μM		CMPD167 5 μM	
Cytokine	FC	*P*	FC	*P*	FC	*P*	FC	*P*	FC	*P*	FC	*P*	FC	*P*
IL-8	3.006	0.00501	1.529	0.05733928	2.120	0.00436993	2.060	0.00076855	1.683	0.02468515	1.757	0.00998727	2.416	0.00279026
MCP-1	3.238	0.087653	1.499	0.15584023	2.187	0.01433895	2.168	0.01726747	1.738	0.06638911	1.644	0.10260723	2.972	0.03720446
IL-6	2.017	0.334037	0.764	0.68371768	1.049	0.9314167	1.038	0.94653174	0.819	0.75175364	0.743	0.65251238	1.299	0.65080303
GCSF	1.504	0.198353	2.008	0.00128579	2.198	0.00064578	2.146	0.00053549	2.055	0.00086059	2.043	0.00116224	2.285	0.00101014
IL-1α	3.862	0.000242	0.102	0.00194046	0.110	0.00203516	0.102	0.00193671	0.101	0.00192632	0.091	0.00182254	0.103	0.00195199
IP-10	4.342	0.025269	20.781	0.01017474	43.274	0.00109754	22.296	0.00291955	23.283	0.00079919	67.610	0.12207202	37.534	0.01205219
IL-1β	2.307	0.189003	1.407	0.14885625	1.746	0.23168245	1.471	0.18394224	1.626	0.12908468	0.922	0.7431148	1.600	0.09848836
IL-16	1.142	0.657738	0.424	0.05508423	0.374	0.04083203	0.398	0.04700847	0.326	0.03016336	1.012	0.96625343	0.356	0.03642212
GMCSF	3.467	0.013994	4.712	0.00086624	6.327	0.00059053	5.038	0.00034721	4.994	0.00013383	8.675	0.00010017	5.626	0.00060681
IL-4	1.082	0.059446	0.920	0.24376849	0.895	0.08667451	0.803	0.01628621	0.828	0.00202642	8.271	3.3093E-10	0.898	0.0062291
IL-2	1.082	0.062902	1.413	6.6863E-05	1.485	4.0905E-05	1.361	0.0002546	1.351	0.00030642	1.737	1.5916E-05	1.492	0.00049214
TGF-β	1.464	0.168631	2.917	0.00469705	3.354	0.00201696	2.750	0.00128127	2.786	0.00122284	43.216	1.5281E-05	3.619	2.0037E-05
RANTES	1.063	0.59086	1.944	0.00040634	2.129	0.00268375	1.785	0.00102752	1.714	0.00115577	5.264	1.8538E-05	2.113	0.00028452
IFN-γ	1.337	0.001258	4.364	8.3403E-06	4.870	1.7025E-08	4.275	1.1104E-06	4.561	1.7943E-05	4.759	0.00011577	5.073	6.0423E-05
IFN-β	1.338	0.11964	1.617	0.03739738	1.967	0.00227115	1.660	0.01091946	1.712	0.00886558	1.307	0.21776557	1.947	0.00616477
TNF-α	1.375	0.071877	2.754	0.00028377	3.548	0.01324633	2.877	0.00296032	3.147	0.00108178	1.506	0.00285317	2.849	0.00016861
MCP-2	4.735	0.067234	51.432	0.05072947	91.411	0.0094317	49.721	0.00670728	43.280	0.00222197	21.968	0.07121319	75.121	0.0670524
IL-12	1.122	0.416508	2.139	1.4441E-05	2.218	0.00015056	2.068	4.9714E-05	2.032	5.3004E-05	2.414	0.00416009	2.141	3.8922E-05
SDF-1β	2.907	0.006027	3.678	4.6573E-05	4.494	0.00141519	3.402	0.00024412	3.961	7.6582E-05	17.695	0.00634484	4.063	8.9628E-05
MIG	4.542	0.031247	9.817	0.07197795	15.825	0.00554168	7.305	0.00920757	10.045	0.00027651	6.370	0.00183757	15.425	0.03740879
IL-15	1.033	0.054584	1.803	3.5785E-09	1.828	6.9092E-08	1.797	1.0511E-08	1.802	9.1126E-09	0.661	0.00096647	1.799	2.3908E-08
MIP-1α	4.008	0.001695	19.311	0.00634913	28.010	0.00550377	14.926	0.00122992	19.808	0.00289946	20.279	0.00453498	22.071	0.00314702
MIP-1β	3.862	0.000242	8.908	0.000907	14.154	0.01166354	8.592	0.00078643	9.773	0.00012989	10.957	0.00171085	11.708	0.00348539

NL4.3 ± ARV, 24 h post-treatment

	Treatment[1]													
	Virus		TFV 35 μM		UC781 12 μM		TMC120 1.2 μM		T1249 10 μM		AMD3465 5 μM		CMPD167 5 μM	
Cytokine	FC	*P*	FC	*P*	FC	*P*	FC	*P*	FC	*P*	FC	*P*	FC	*P*
IL-8	1.401	0.0174670	0.380	0.0004580	0.379	0.0001635	0.827	0.5454217	0.442	0.0084902	0.509	0.1100005	0.530	0.0074939
MCP-1	1.896	0.3285710	0.366	0.2957276	0.376	0.3019378	0.824	0.7722618	0.484	0.3962649	0.396	0.3250243	0.738	0.6626601
IL-6	1.492	0.3624030	0.239	0.0282220	0.227	0.0260925	0.521	0.1445797	0.314	0.0472173	0.341	0.0767192	0.446	0.0937537
GCSF	1.368	0.5790030	0.356	0.2310158	0.355	0.2295146	0.819	0.7300002	0.466	0.3172024	0.438	0.2958473	0.594	0.4411945
IL-1α	2.480	0.0051920	0.032	0.0002665	0.022	0.0002509	0.028	0.0002599	0.031	0.0002644	0.036	0.0002719	0.028	0.0002603
IP-10	3.877	0.0055860	2.899	0.0190658	5.160	0.0026196	5.993	0.0010067	3.816	0.0070745	3.469	0.0112389	4.189	0.0021086
IL-1β	4.501	0.0014490	0.995	0.9860854	0.578	0.0273499	0.787	0.1509196	0.896	0.5679274	1.329	0.2821959	0.671	0.1357376
IL-16	1.988	0.0397330	0.722	0.3828574	0.912	0.7374762	0.834	0.4831560	0.856	0.5525870	1.060	0.8097070	0.603	0.1455358
GMCSF	1.312	0.5101760	0.346	0.0925471	0.396	0.1186114	0.634	0.3685488	0.418	0.1314363	0.401	0.1441656	0.546	0.2294943
IL-4	0.960	0.5023540	0.772	0.0986937	0.891	0.4271575	0.853	0.3059482	0.815	0.0272299	0.963	0.6827788	0.728	0.0184228
IL-2	1.191	0.0011090	0.293	0.0000004	0.363	0.0000066	0.320	0.0000007	0.346	0.0000005	0.346	0.0000004	0.338	0.0000130
TGF-β	1.492	0.1932830	10.365	0.0000740	12.039	0.0001273	12.670	0.0000762	11.025	0.0000002	10.692	0.0000017	12.212	0.0010466
RANTES	2.815	0.0000250	3.308	0.0102562	3.903	0.0009570	4.736	0.0023954	3.643	0.0003587	3.934	0.0370814	9.917	0.0020720
IFN-γ	1.982	0.0028020	3.485	0.0000771	3.843	0.0013911	4.053	0.0001173	3.841	0.0000000	3.923	0.0000994	4.134	0.0007759
IFN-β	1.352	0.4113200	0.827	0.5479382	0.856	0.6350494	0.938	0.8358543	0.791	0.4550187	0.891	0.7110336	0.891	0.7029955
TNF-α	0.802	0.6050640	1.080	0.8320222	1.144	0.7077689	1.090	0.8123863	1.084	0.8233903	1.114	0.7633241	1.048	0.8981908
MCP-2	2.455	0.2031060	0.640	0.5562800	0.944	0.9306180	2.208	0.2880729	0.932	0.9154170	1.284	0.7970907	1.460	0.5731367
IL-12	0.849	0.3861990	1.991	0.0280854	2.191	0.0195625	2.261	0.0050894	2.120	0.0018222	2.144	0.0014094	2.366	0.0323476
SDF-1β	0.961	0.8926440	0.663	0.3204186	0.924	0.8404369	0.910	0.7925027	0.576	0.1608611	0.779	0.5209030	1.005	0.9912996
MIG	2.743	0.0034150	2.656	0.0143274	2.709	0.0146998	4.015	0.0278270	3.074	0.0361457	2.294	0.1717037	4.242	0.0032220
IL-15	0.996	0.9086240	0.784	0.0002704	0.777	0.0002198	0.775	0.0003466	0.776	0.0002565	0.788	0.0008620	0.778	0.0003862
MIP-1α	2.039	0.0005010	0.894	0.7355809	1.240	0.4266070	1.337	0.3961376	0.885	0.4915297	1.375	0.4053038	1.844	0.0240198
MIP-1β	1.906	0.0003610	0.880	0.5566051	0.777	0.1851834	0.921	0.6665062	0.898	0.5177726	1.197	0.6326865	1.098	0.7790910

YU.2 ± ARV, 24 h post-treatment

	Treatment[1]													
	Virus		TFV 35 μM		UC781 12 μM		TMC120 1.2 μM		T1249 10 μM		AMD3465 5 μM		CMPD167 5 μM	
Cytokine	FC	*P*	FC	*P*	FC	*P*	FC	*P*	FC	*P*	FC	*P*	FC	*P*
IL-8	1.337	0.0389870	0.473	0.0011165	0.410	0.0012650	0.612	0.0659179	0.556	0.0012095	0.575	0.0028104	0.712	0.0674364
MCP-1	1.213	0.7172410	0.352	0.2829778	0.370	0.2997611	0.819	0.7661927	0.607	0.5121328	0.565	0.4613420	0.649	0.5556887
IL-6	1.331	0.5313170	0.308	0.0495420	0.441	0.1161348	0.618	0.2791819	0.473	0.1029400	0.435	0.0799426	0.481	0.1127375
GCSF	1.021	0.9709800	0.409	0.2692106	0.453	0.3048207	0.829	0.7442351	0.673	0.5302501	0.611	0.4534366	0.715	0.5869216
IL-1α	1.756	0.0414550	0.090	0.0003861	0.046	0.0002881	0.088	0.0003759	0.057	0.0003105	0.065	0.0003231	0.056	0.0003073
IP-10	1.912	0.2657580	1.807	0.0855622	2.540	0.0081892	2.681	0.0134881	2.607	0.1155145	13.479	0.0001061	2.585	0.0261995
IL-1β	2.417	0.0066270	6.429	0.0454565	1.926	0.3618078	6.798	0.0124335	2.820	0.2139008	2.700	0.0603590	2.396	0.2224539
IL-16	1.362	0.3189360	1.047	0.8488210	0.998	0.9962949	1.232	0.3834499	1.074	0.8327936	0.905	0.7867994	1.040	0.8652824
GMCSF	0.863	0.7367920	1.093	0.8167714	1.164	0.7225470	1.182	0.6857004	1.501	0.3689753	1.495	0.2779533	1.268	0.5157069
IL-4	0.928	0.2759560	0.921	0.4309068	0.930	0.3807783	1.157	0.1452495	0.988	0.8903432	7.059	0.0000000	0.951	0.6250305
IL-2	0.985	0.5803690	1.390	0.0000100	1.439	0.0000020	1.473	0.0001836	1.413	0.0000555	1.650	0.0000004	1.479	0.0000375
TGF-β	0.891	0.7541950	1.460	0.1984401	1.703	0.0650697	1.824	0.0693413	1.861	0.1645517	15.462	0.0000090	1.723	0.1038772
RANTES	1.504	0.0758560	1.773	0.0026096	2.149	0.0114397	1.944	0.0145363	2.553	0.0518254	13.256	0.0060641	1.925	0.0171040
IFN-γ	1.247	0.0058610	3.580	0.0001059	3.913	0.0000585	4.614	0.0000549	4.434	0.0012683	4.920	0.0000011	4.614	0.0003318
IFN-β	1.069	0.8200220	1.082	0.7848671	1.219	0.4989762	1.455	0.2998750	1.581	0.2502435	0.987	0.9608318	1.431	0.2153864
TNF-α	0.642	0.3627100	2.934	0.0148127	1.522	0.2557333	2.771	0.0544393	2.162	0.0380437	0.948	0.8920477	2.117	0.1785339
MCP-2	0.580	0.5141360	3.101	0.1069450	3.776	0.0451047	3.853	0.1614117	6.990	0.0751679	4.375	0.0782068	5.522	0.0140242
IL-12	0.732	0.1528990	1.568	0.0185650	1.400	0.0477778	1.687	0.0101278	1.538	0.0438310	1.743	0.0270627	1.407	0.0567381
SDF-1β	0.621	0.2524090	1.085	0.7798560	0.748	0.3843408	1.297	0.3501456	1.029	0.9232182	3.064	0.0227383	0.912	0.7749842
MIG	1.121	0.7384060	2.437	0.0329276	3.168	0.0353016	3.294	0.0732195	4.191	0.0793329	2.186	0.0689414	3.912	0.0119486
IL-15	0.943	0.1210740	1.659	0.0000005	1.632	0.0000011	1.712	0.0000062	1.691	0.0000395	0.835	0.0321624	1.684	0.0000057
MIP-1α	1.372	0.0223350	4.752	0.0296481	2.035	0.0068486	3.459	0.0021814	4.643	0.0028506	2.817	0.0352816	4.536	0.0620541
MIP-1β	1.200	0.3449600	2.825	0.0118524	1.708	0.0637622	2.683	0.0071793	3.314	0.0307902	2.687	0.1860332	2.960	0.0172630

[1]Significantly modulated cytokines as determined by unpaired *t* test with no correction for multiple testing ($P < 0.05$). FC: Fold change
