# Supplementary material for: Early Colorectal Responses to HIV-1 and Modulation by Antiretroviral Drugs
Source: Vaccines (Basel). 2021 Mar 8;9(3):231. doi: 10.3390/vaccines9030231 (PMC8000905; doi:10.3390/vaccines9030231)
Supplement: Supplementary file 1 [file vaccines-09-00231-s001.zip › Supplementary Figure 2 new.pdf]

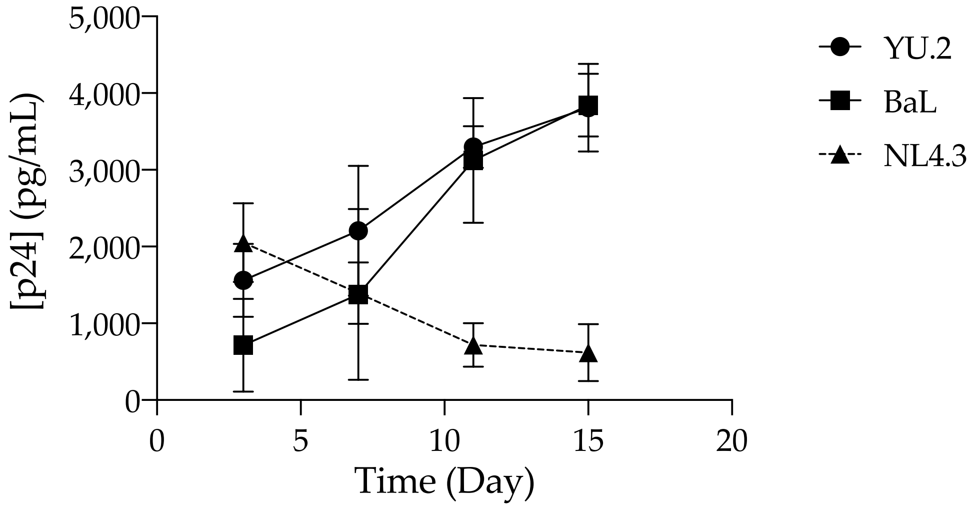

Figure S2. Kinetics of YU.2, BaL and NL4.3 replication in human colorectal explants. Colorectal explants were incubated with YU.2 (●), PA-GFP-VPR BaL (■), or NL4.3 (▲), for 2 h, washed four times in PBS, and cultured on gel foam for 15 days. Supernatants were harvested at on days 3, 7, 11, and 15, and the cultures were refed with fresh medium. Levels of p24 concentrations in culture supernatants were measured by ELISA (Innotest HIV antigen kit, Innogenetics, Belgium) according to the manufacturer's instructions. Data are means (± SD) of at least two independent experiments performed in triplicate.
